# Comparing the efficacy of different types of exercise for the treatment and prevention of depression in youths: a systematic review and network meta-analysis

**DOI:** 10.3389/fpsyt.2023.1199510

**Published:** 2023-06-02

**Authors:** Yihan Zhang, Geng Li, Chengzhen Liu, Jinliang Guan, Yuantong Zhang, Zifu Shi

**Affiliations:** ^1^School of Educational Science, Hunan Normal University, Changsha, China; ^2^College of Physical Education, Hunan Normal University, Changsha, China

**Keywords:** depression, anxiety, exercise, youth, treatment, prevention

## Abstract

**Purpose:**

Depression disorder is the most commonly diagnosed type of mental illness among youths. Although a plethora of evidence suggests a positive relationship between exercise and lower levels of depression in youths, the findings regarding the variation in magnitude of this relationship are inconclusive with respect to the preventive and therapeutic effects of different types of exercise. This network meta-analysis aimed to determine the best type of exercise for the treatment and prevention of depression in youths.

**Methods:**

A comprehensive search of databases, including PubMed, EMBASE, The Cochrane Library, Web of Science, PsychINFO, ProQuest, Wanfang, and CNKI, was conducted to identify relevant research on exercise interventions for depression in youth populations. The risk of bias in the included studies was evaluated using Cochrane Review Manager 5.4 according to the Cochrane Handbook 5.1.0 Methodological Quality Evaluation Criteria. The network meta-analysis was performed using STATA 15.1 to calculate the standardized mean difference (SMD) of all concerned outcomes. The node-splitting method was used to test the local inconsistency of the network meta-analysis. Funnel plots were used to evaluate the potential impact of bias in this study.

**Result:**

Utilizing data extracted from 58 studies (10 countries, 4,887 participants), we found that for depressed youths, exercise is significantly better than usual care in reducing anxiety (SMD = −0.98, 95% CI [-1.50, −0.45]). For non-depressed youths, exercise is significantly better than usual care in reducing anxiety (SMD = −0.47, 95% CI [ −0.66, −0.29]). In the treatment of depression, resistance exercise (SMD = −1.30, 95% CI [ −1.96, −0.64]), aerobic exercise (SMD = −0.83, 95% CI [-1.10 −0.72]), mixed exercise (SMD = −0.67, 95% CI [−0.99, −0.35]), and mind-body exercise (SMD = −0.61, 95% CI [−0.84, −0.38]) all showed significant efficacy over usual care. For the prevention of depression, resistance exercise (SMD = −1.18, 95% CI [-1.65, −0.71]), aerobic exercise (SMD = −0.72, 95% CI [−0.98, −0.47]), mind-body exercise (SMD = −0.59, 95% CI [-0.93, −0.26]), and mixed exercise (SMD = −1.06, 95% CI [−1.37 to −0.75]) were all significantly effective compared to usual care. According to the test of the surface under the cumulative ranking score (SUCRA), the ranking of exercises for the treatment of depression in depressed youths is as follows: resistance exercise (94.9%) > aerobic exercise (75.1%) > mixed exercise (43.8%) > mind-body exercise (36.2%) > usual care (0%). For the prevention of depression in non-depressed youths, resistance exercise (90.3%) > mixed exercise (81.6%) > aerobic exercise (45.5%) > mind-body exercise (32.6%) > usual care (0%). Resistance exercise thus had the best comprehensive effect on both the treatment and prevention of depression in youths (clusterank value = 1914.04). Subgroup analyses show that a frequency of 3–4 times per week, a duration of 30–60 min, and a length of more than 6 weeks were found to be the most effective interventions for depression (*P* > 0.001).

**Conclusion:**

This study provides compelling evidence that exercise is a viable intervention for improving depression and anxiety in young individuals. In addition, the study emphasizes the importance of selecting the appropriate type of exercise to optimize treatment and prevention. Specifically, the results suggest that resistance exercise, performed 3–4 times per week, with sessions lasting 30–60 min and a length of more than 6 weeks, yields optimal results for the treatment and prevention of depression in young individuals. These findings have significant implications for clinical practice, particularly given the challenges associated with implementing effective interventions and the economic burden of treating and preventing depression in young people. However, it is worth noting that additional head-to-head studies are necessary to confirm these findings and strengthen the evidence base. Nevertheless, this study provides valuable insights into the role of exercise as a potential treatment and preventative measure for depression in young people.

**Systematic review registration:**

https://www.crd.york.ac.uk/PROSPERO/display_record.php?RecordID=374154, identifier: 374154.

## 1. Introduction

Depression is a mood disorder characterized by persistent feelings of sadness, loss of interest in activities, and other symptoms that impair daily functioning (1). It has become a significant public health issue for today's youth (2, 3). During the period of transition from adolescence to adulthood, which typically ranges from 15 to 24 years of age (4), individuals are psychologically underdeveloped and have an increased risk for depression (5, 6). The deleterious effects of depression can extend to a youth's social relationships (7) and academic performance (8) and can even lead to suicidal ideation (9). In most countries, clinical interventions for depression primarily involve psychotherapy and medication (10). However, despite the adequate implementation of such treatments, a significant portion of patients do not experience sufficient symptom relief, with 50% experiencing at least one new depressive episode after 6–12 months of treatment (11). Although psychotherapy can be effective, it can also be expensive and stigmatizing (12). In addition, despite their potential benefits, there is substantial evidence to suggest that antidepressant medications can have adverse effects including an increased risk of suicidal behavior (13) and a high rate of discontinuation among patients, with 20 to 59% discontinuing their medication within 3 weeks (14, 15). Therefore, it is crucial to implement cost-effective and robust interventions to establish recovery and prevent relapse of depression in youths.

In recent years, there has been growing interest in the use of physical exercise as a treatment for depression or as an adjunct to traditional therapies (16). In this context, “physical exercise” refers to planned, structured, repetitive, and purposeful physical activities aimed at improving or maintaining one or more of the components of physical fitness (17). Research to date has suggested that exercise has promising antidepressant effects as it increases the production of monoamines such as dopamine and serotonin, which are crucial for the treatment of depression (18–20). Exercise-induced changes in neuroplasticity and brain-derived neurotrophic factor (BDNF) may also contribute to its antidepressant effects (21). A growing body of cross-sectional studies has shown that inadequate physical activity is a significant risk factor for depression (22). For these reasons, numerous randomized controlled trials (RCTs) have been conducted and found to show that structured exercise programs can effectively alleviate depression (23, 24). For example, an 8-week aerobic exercise intervention improved depression in depressed patients by improving their cognitive control function and rumination patterns (25). Similarly, a 20-week RCT found that depression was significantly reduced by resistance training, and the effect persisted for 26 months after the end of the intervention (26). Mind-body exercise has also been found to be effective in reducing depression, anger, and fatigue (27). Moreover, mixed exercise interventions have been found to improve cardiorespiratory fitness and significantly improve depression and anxiety (28). Furthermore, systematic reviews and meta-analyses have reported moderate-to-large antidepressant effects of exercise interventions in different age groups, including children (29), adolescents (30), young adults 13), and older adults (31). Although exercise is now known to be generally beneficial for depression, different types of exercise elicit different physical mechanisms (32). It is hypothesized, therefore, that different types of exercise may have varying degrees of effectiveness in treating depression. While numerous meta-analyses have assessed the efficacy of exercise interventions, uncertainty remains over the relative efficacy of different exercise types in treating depression.

If the development of depression is uncontrolled during youth, it often becomes chronic in adulthood (33). Therefore, the early effective prevention of depression in youths is crucially important. Moreover, physical exercise has a protective effect against the development of depression (34). This is because, first, exercise may cause changes in the levels of various neurotransmitters in the brain, such as serotonin, dopamine, and norepinephrine. These neurotransmitters play important roles in regulating mood and alterations in their levels can affect the development of depression. Second, exercise can also trigger the release of endorphins, which are natural opioids produced by the body that can reduce pain and promote feelings of wellbeing and euphoria; they also play a role in preventing depression. Third, engaging in exercise can provide opportunities for social interaction and support, which can also have a positive impact on mental health and the prevention of depression. Overall, these mechanisms highlight the importance of exercise for improving the physiological and psychological factors associated with depression and suggest that exercise has both direct and indirect effects in preventing depression. While Rebars' meta-meta-analyses further support the preventive efficacy of exercise, uncertainty remains over the relative preventive efficacy of different exercise types in depression.

Anxiety is an excessive concern, unease, and fear about specific things or situations (1); it is frequently co-morbid with depression (35). Feelings of anxiety can interfere with an individual's ability to carry out daily activities and can lead to a reduced quality of life (36). In fact, anxiety and depression share many symptoms and thus can be difficult to differentiate (35). Prior studies have demonstrated that the co-occurrence of anxiety and depression is associated with a higher risk of developing more severe symptoms (37), increased rates of disability, and reduced response to treatment (38). However, several studies have demonstrated that exercise has favorable effects on anxiety (39). Individuals experiencing anxiety often report bodily tension and discomfort (36). Exercise has been shown to dissipate excess energy, alleviate muscular tension (40), and enhance metabolic processes (41), leading to a reduction in anxiety (42). While meta-analyses have found that exercise is effective in improving anxiety (39), uncertainty remains about whether exercise has different effects on anxiety in depressed vs. non-depressed youths. Therefore, it is necessary to examine the effect of exercise intervention on improving anxiety in depressed vs. non-depressed youths to scrutinize the mechanism by which exercise affects depression.

Meta-analyses have limitations when comparing multiple interventions as pooling individual trial effects may prevent such comparisons (43). However, network meta-analysis (NMA) is a commonly employed methodology to integrate evidence across multiple studies with varying interventions. By circumventing the assumption of homogeneity in treatments (44), network meta-analysis mitigates potential biases stemming from small study effects and accounts for the inherent variations in the control group. Furthermore, network meta-analysis can comprehensively rank the effectiveness of interventions on two outcomes simultaneously, which renders it a more suitable option in the current research context for evaluating the comparative efficacy of diverse types of exercise (45–47). Miller et al. (43) network meta-analysis has provided compelling evidence for the effectiveness of different exercise interventions in ameliorating depression in older adults. However, an exploration of the comparative effectiveness of different types of exercise and the comprehensive rankings of those types of exercise for the treatment and prevention of depression in youths have not yet been undertaken.

Given that this is the case, this study seeks to answer four specific research questions: (i) which type of exercise provides the best therapeutic effect for depression in youths? (ii) Which type of exercise provides the best preventive effect for depression in youths? (iii) Does exercise have a positive impact on anxiety levels in both depressed and non-depressed youths? Additionally, the study aims to investigate (iv) whether certain types of exercise can be combined for maximum therapeutic and preventive effects for depression in youths. Answering these questions could contribute to the development of more effective exercise interventions for depression and anxiety in youths.

## 2. Methods

The Preferred Reporting Items for Systematic Reviews and Meta-Analyses (PRISMA) statement was used to report this systematic review and network meta-analysis (48). This review was registered in the PROSPERO international prospective register of systematic reviews (CRD42022374154).

### 2.1. Search strategy

The authors GL and CL performed a systematic search using a search strategy based on the Cochrane Handbook of Systematic Reviews (49) and the PICOS principle.

Participants: youths aged 15–24 years.

Interventions: aerobic exercise, resistance exercise, mind-body exercise, and mixed exercise.

Comparisons: usual care including daily care, waitlist control conditions, placebo, or other social activities.

Outcomes: depression and anxiety in depressed or non-depressed youths were measured by structured clinical interviews using established diagnostic criteria and validated using above-threshold depressive symptoms screening measures. Scales for measuring depression included the Beck Depression Inventory (BDI), the Patient Health Questionnaire (PHQ-9), the Center for Epidemiologic Studies Depression Scale (CES-D), and the Hamilton Rating Scale for Depression (HAM-D). Scales for measuring anxiety included the Hamilton Anxiety Rating Scale (HAM-A), the Beck Anxiety Inventory (BAI), and the Zung Self-Rating Anxiety Scale (SAS). More information about the established diagnostic criteria and scales used in the included studies can be found in the Supplementary material.

Study: randomized controlled trials (RCTs).

The search databases included PubMed, EMBASE, The Cochrane Library, Web of Science, PsychINFO, ProQuest, Wanfang, and CNKI. The corresponding search strategies were tailored to the characteristics of each database. In order to guarantee the comprehensiveness and precision of our network meta-analysis, we did not restrict searches by year of publication or language and implemented a meticulous data acquisition methodology. Specifically, the reference lists for each coded full-text article were examined to identify additional eligible studies. We endeavored to establish communication with the corresponding authors of studies that satisfied the predetermined eligibility standards but lacked essential data in an effort to procure the requisite information. All studies included in the analysis were collected and coded in November 2022. During the search process, the lists of articles were revised and finalized based on the discussion and evaluation. The detailed search strategy, the definitions of variables, and the assumptions and simplifications used in this study are provided in the Supplementary material.

### 2.2. Exclusion criteria

This study excluded: (1) non-randomized controlled trials, case reports, physician experiences, book reports, and literature reviews; (2) animal studies; (3) purely descriptive studies; (4) repeated data studies; (5) studies with an unclear diagnosis of depression or other co-morbidities; (6) studies with unclear results, incomplete data, or unsuccessful contact with full-text authors; (7) studies with unclear definitions of the types of exercise included; (8) exercise combined with drug interventions.

### 2.3. Study selection

NoteExpress literature management software was utilized to efficiently handle the downloaded literature data by detecting duplicate titles and merging the outcomes of literature searches from the various databases. This helped to create an information database and facilitated a full-text download of the studies. Subsequently, in line with the inclusion and exclusion criteria, two researchers independently evaluated the titles and abstracts of the studies. Following this initial assessment, the researchers read the complete texts to determine whether to exclude them. Relevant information was extracted from any studies meeting the inclusion criteria. Lastly, the two researchers cross-checked their screening outcomes. Any differences found were discussed to arrive at a resolution, or a third researcher was consulted to resolve the disagreement.

### 2.4. Data extraction

To assess the effectiveness of exercise interventions in improving depression, we initially categorized the included studies into depressed and non-depressed groups using screening measures of the structured clinical interviews, established diagnostic criteria, and validated above-threshold depressive symptoms. For example, a score on the Beck Depression Inventory-II (BDI-II) >13 was depressed groups, while a score on the Self-rated Depression Scale (SDS) >50 was depressed groups. Subsequently, by comparing changes in depression scores between the exercise intervention group and the control group that did not receive any exercise intervention, we could determine whether the exercise intervention had a positive impact on alleviating depression.

The study extracted the following information from the literature: (1) essential details regarding the included studies such as the first author, journal, year of publication, and study title; (2) information concerning the experimental and control groups featured in each study, including the number of experimental and control groups, participants' ages, and outcome measures; (3) information about the study design and quality assessment of the included studies; (4) data on the outcomes measured; (5) information on the interventions used in the experimental group, including the type of exercise, duration, frequency, and length of the intervention.

### 2.5. Risk of bias in individual studies

The assessment of bias was conducted using Cochrane Review Manager 5.4 in accordance with the Methodological Quality Evaluation Criteria outlined in the Cochrane Handbook 5.1.0. The studies included in the analysis were evaluated on six metrics, including the random assignment method, allocation concealment, blinding, completeness of the outcome data, selective reporting of study results, and other sources of bias. The authenticity of the study outcome report and any other potential sources of bias were also evaluated. Each metric was categorized as having a high, low, or uncertain risk of bias. The risk of bias was evaluated qualitatively based on the descriptions provided in the included studies.

### 2.6. Statistical analysis

The present study utilized the “network” package in Stata 15.1 software to perform a network meta-analysis based on the frequency framework. This method efficiently handled data from multi-arm trials, enabling comprehensive comparisons across multiple interventions while maintaining statistical power and precision of the estimates (44). Given that the outcome indicators were continuous variables measured on different scales, the effect size indicators used were the standardized mean difference (SMD) and the 95% confidence interval (CI). To ensure the appropriateness of the statistical model, we assessed model fit and heterogeneity using the Q test and I^2^ in the forest plot, with statistical significance set at *p* < 0.05. The appropriate variance structure was determined from I^2^, with the fixed-effects model applied when I^2^ was < 50% and the random-effects model applied when I^2^ was ≥50% (50). Inconsistency in closed loops of network plots was investigated using the node-splitting method, with a loop inconsistency factor (IF) lower than or close to zero taken to indicate good agreement between direct and indirect evidence (51).

Intervention rankings were determined by comparing the surface under the cumulative ranking score (SUCRA), with a higher SUCRA value taken to indicate a higher probability of ranking (52). A two-dimensional cluster ranking plot was constructed based on the SUCRA values of different exercise types used for the treatment and prevention of depression to determine the best suitable choice for youths. Subgroup analysis and meta-regression analysis were used to explore the underlying moderators. A funnel plot was used to determine whether there was a small sample effect between the studies. Sensitivity analysis was performed to explore sources of heterogeneity, such as removing studies with a high risk of bias or evaluating the impact of different statistical models on the results (50). Overall, exploring the geometry of the treatment network and potential biases by the above methods helped to ensure the validity and reliability of the network meta-analysis results.

## 3. Results

### 3.1. Literature selection

**Figure 1:**
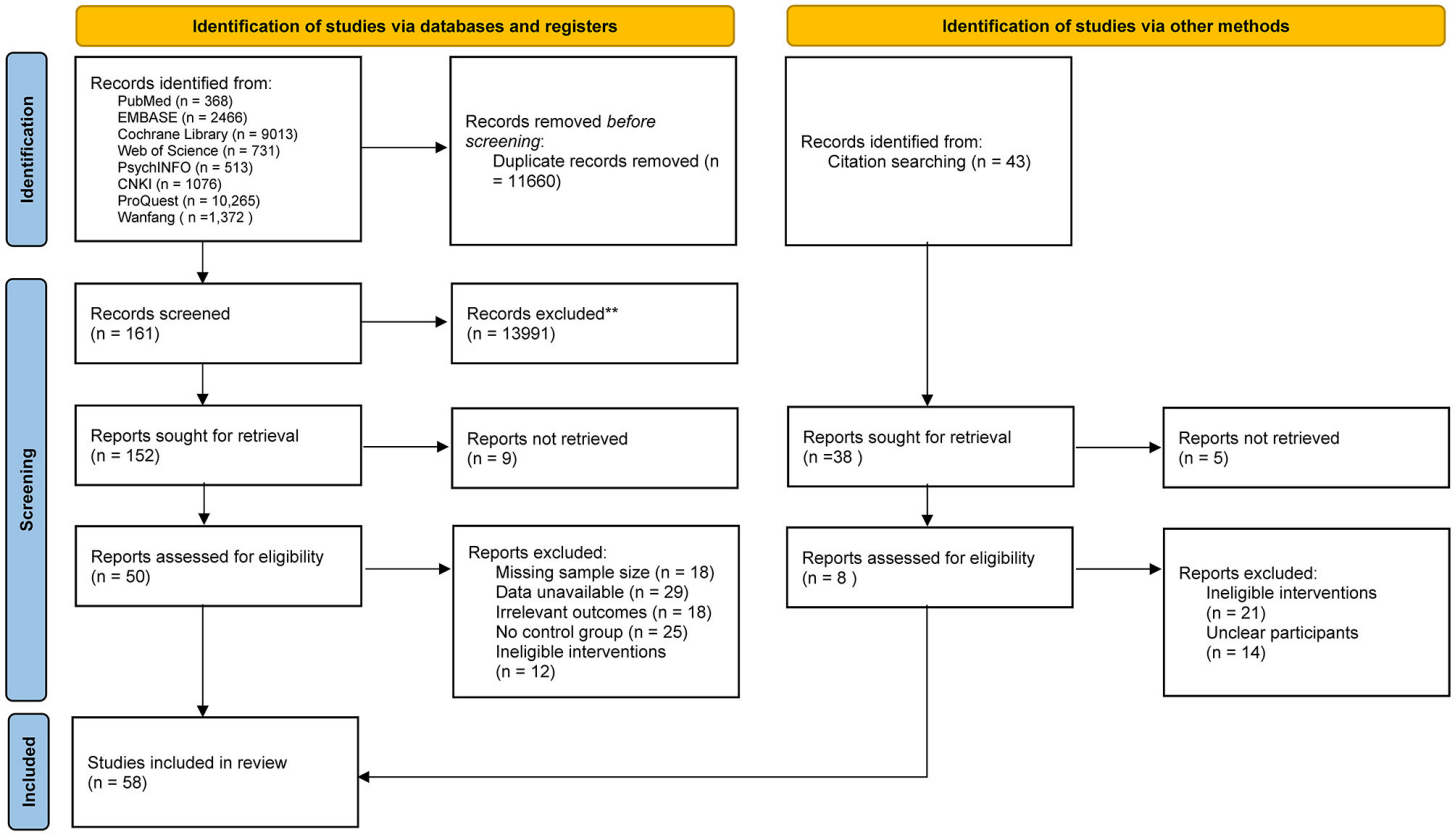
PRISMA flow diagram.

The initial search of the literature yielded 25,804 references, of which 368 were from PubMed, 2,466 were from EMBASE, 9,013 were from The Cochrane Library, 731 were from Web of Science, 513 were from PsychINFO, 1,076 were from CNKI, 10,265 were from ProQuest, 1,372 were from Wanfang, and 43 were from citation searches. This selection was independently screened and re-screened by two researchers according to the inclusion and exclusion criteria. Ultimately, 58 articles were obtained (Figure 1).

### 3.2. Characteristics of the included studies

The above-described 58 academic papers consisted of studies involving 4,887 participants from 10 distinct countries. The average age of the participants ranged from 15 to 22 years old, and they were randomly assigned to either an experimental or control group. The studies investigated the effects of four exercise interventions including aerobic, resistance, mind-body, and mixed exercises on depression and anxiety scores among depressed and non-depressed youths. More detailed information about the studies can be found in Table 1.

**Table 1:** League table for head-to-head comparisons.

**Resistance**	**−0.46 (−0.96, 0.04)**	**−0.12 (−0.68, 0.44)**	−0.59 (−1.16, −0.01)^*^	−1.18 (−1.65, −0.71)^*^
−0.39 (−1.06, 0.28)	Aerobic	0.34 (−0.05, 0.72)	−0.13 (−0.52, 0.26)	−0.72 (−0.98, −0.47)^*^
−0.63 (−1.36, 0.10)	−0.24 (−0.60, 0.12)	Mixed	−0.47 (−0.92, −0.01)^*^	−1.06 (−1.37, −0.75)^*^
−0.69 (−1.38, 0.01)	−0.30 (−0.59, 0.00)	−0.06 (−0.45, 0.34)	Mind-body	−0.59 (−0.93, −0.26)^*^
−1.30 (−1.96, −0.64) ^*^	−0.91 (−1.10, −0.72)^*^	−0.67 (−0.99, −0.35)^*^	−0.61 (−0.84, −0.38)^*^	Usual care

Depression of non-depressed youth (upper) and Depression of depressed youth (lower) are reported as Hedges'g and 95% confidence intervals.

* *p* > 0.05.

### 3.3. Results of risk of bias assessment

**Figure 2:**
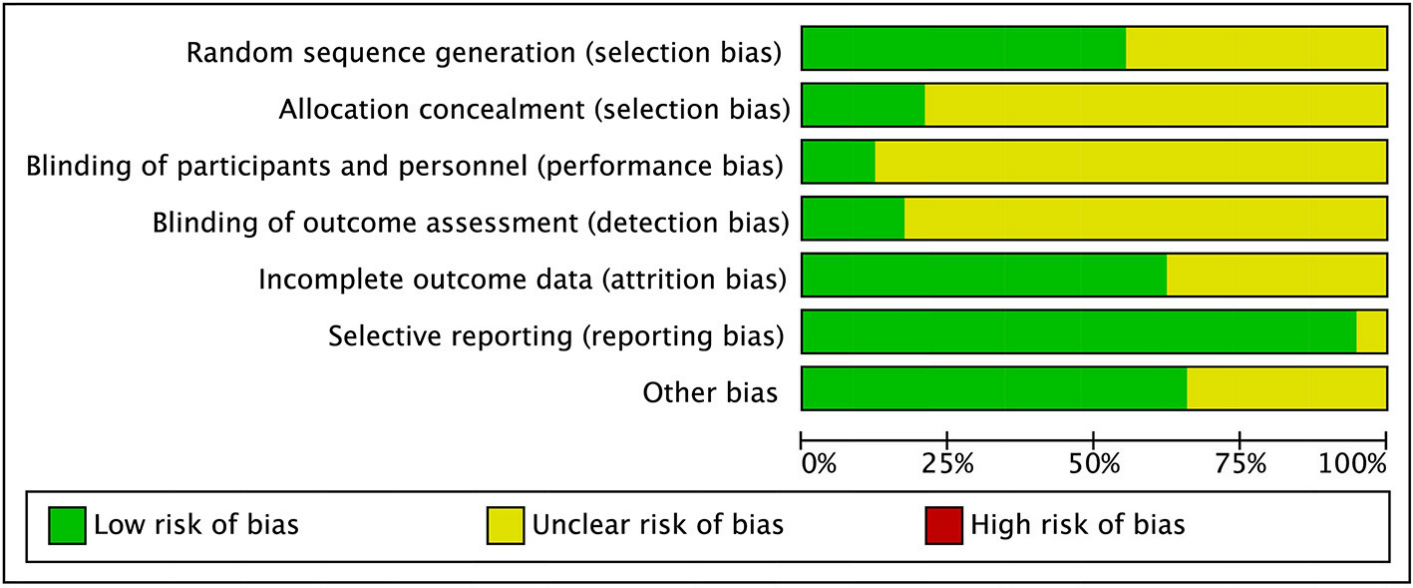
Risk of bias graph.

Of the 58 included studies (see Figure 2), 32 studies had a low risk of bias for random sequence generation and the remaining 26 studies did not describe the allocation method in detail. Twelve studies indicated concealment of allocation. Most studies did not adopt a double-blind experimental protocol because the characteristics of the exercise intervention made it difficult to use blinding of the participants and main subjects (53). There was a low risk of bias for incomplete outcomes in 36 of the 58 studies, and detailed information on the risk of bias for the included studies is available in the Supplemental material.

### 3.4. Anxiety

Due to the limited availability of studies including anxiety indicators in both depressed and non-depressed youths, only a meta-analysis was performed (see Supplemental material).

#### 3.4.1. Anxiety in depressed youths

Three studies with a total of 316 participants, 165 in the experimental group and 151 in the control group, were included in this part of the study. The meta-analysis results showed relatively large heterogeneity with I^2^ = 64.8, indicating the need for a randomized model. The combined-effects result was SMD = −0.98, 95% CI [−1.50, −0.45], indicating that exercise had a significant effect on reducing anxiety in depressed youths compared to usual care.

#### 3.4.2. Anxiety in non-depressed youths

Eleven studies with 1,352 participants, 720 in the experimental group and 632 in the control group, were included in this part of the study. The meta-analysis revealed a relatively large heterogeneity with I^2^ = 53.1, indicating the need for a randomized model. The combined-effects result was SMD = −0.47, 95% CI [−0.66, −0.29], indicating that exercise had a significant effect on reducing anxiety in non-depressed youths compared to usual care.

### 3.5. Depression

**Figure 3:**
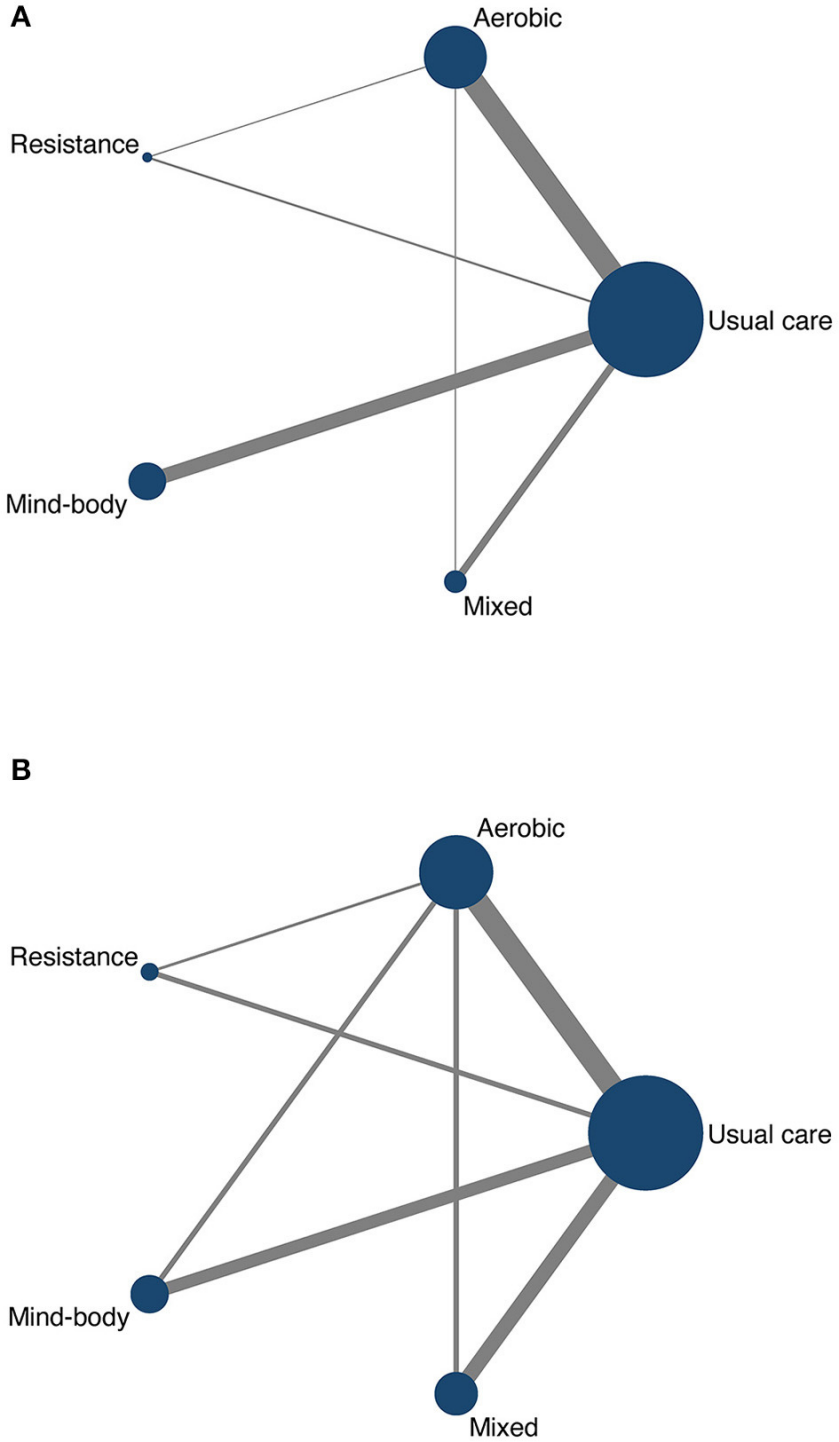
Network plot of depressed youths **(A)** and non-depressed youths **(B)**.

**Figure 4:**
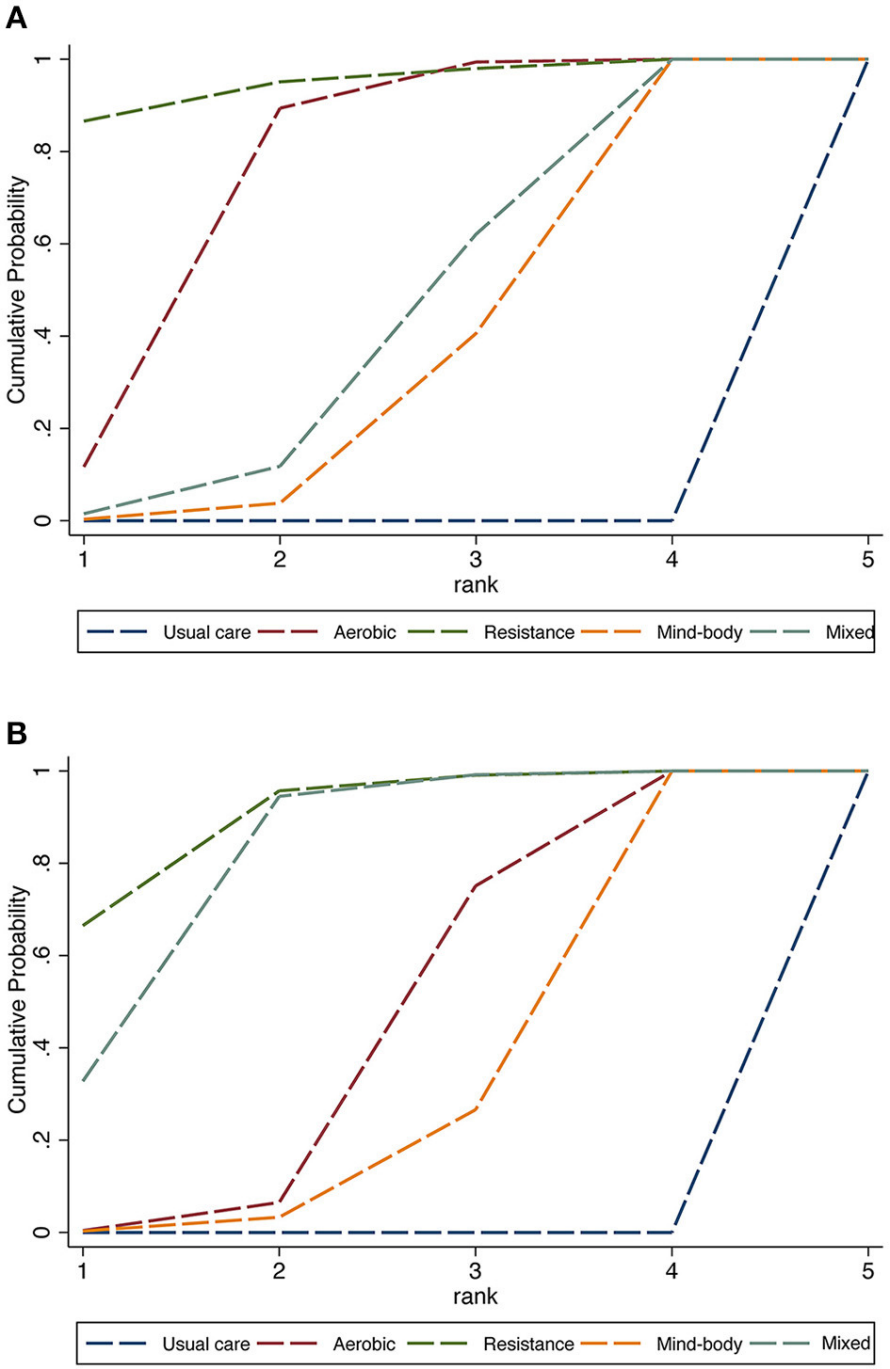
Rank probability of interventions in depressed youths **(A)** and non-depressed youths **(B)**.

**Figure 5:**
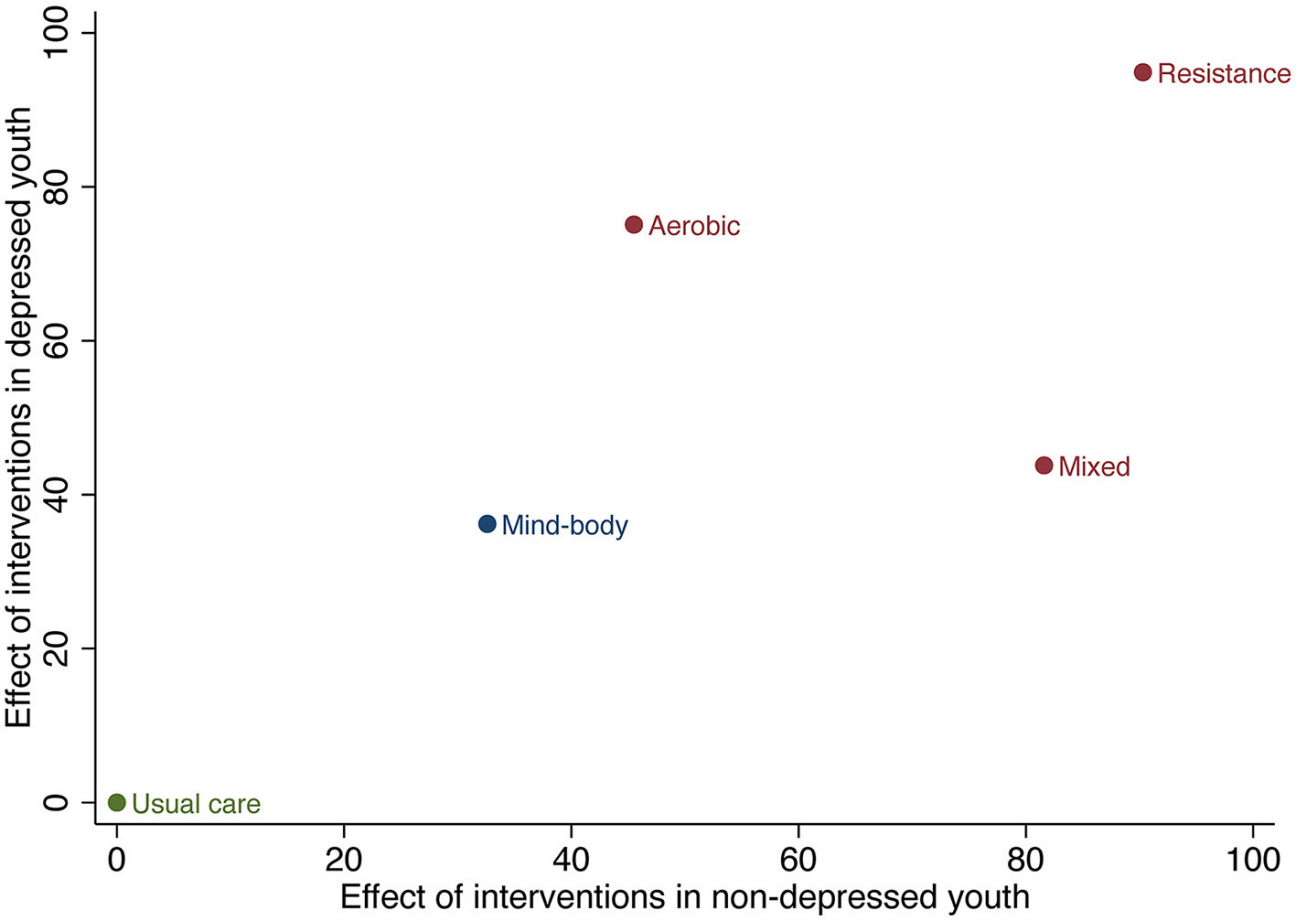
Two-dimensional cluster ranking plot of interventions in depressed youths and non-depressed youths.

All network meta-analyses in this study share the principles of coherence, transferability, and consistency. Figure 3 presents the studies included in this study. Specifically, the size of the nodes reflects the number of participants for that type of intervention, and the thickness of the lines linking interventions reflects the number of studies that compare those interventions. Table 1 shows the head-to-head comparisons between different types of exercise and usual care. Figure 4 shows the rankings of interventions according to their likelihood of being the best choice for non-depressed or depressed youths. Figure 5 illustrates the combined benefits of different interventions in terms of their therapeutic and preventive effects.

#### 3.5.1. Depression in depressive youths

Forty-one studies, covering 2668 participants and four types of exercise contributed to the NMA used to assess depression in depressed youths. The network relationship was centered on usual care and formed three closed loops (see Figure 3A). We performed local inconsistency tests on the closed loops using the node-splitting method; all passed the test, and thus further network meta-analysis was performed. The analysis showed IF values in the range of 0.02 to 0.33, indicating no significant inconsistency in the closed loops.

The head-to-head comparisons are shown in Table 1, where different interventions are compared individually with usual care. However, resistance exercise (SMD = −1.30, 95% CI [−1.96, −0.64]), aerobic exercise (SMD = −0.83, 95% CI [−1.10, −0.72]), mixed exercise (SMD = −0.67, 95% CI [−0.99, −0.35]), and mind-body exercise (SMD = −0.61, 95% CI [−0.84, −0.38]) were all significantly effective in decreasing depression when compared to the usual care received by the control group. Finally, the area under the SUCRA was used to rank the four exercise intervention types and usual care. As can be seen in Figure 4A, the results show that resistance exercise (94.9%) > aerobic exercise (75.1%) > mixed exercise (43.8%) > mind-body exercise (36.2%) > usual care (0%).

#### 3.5.2. Depression in non-depressed youths

Seventeen studies, covering 2219 participants and four types of exercise contributed to the NMA assessing the depression level in non-depressed youths. The network relationship was centered on usual care and formed four closed loops, as shown in Figure 3B. We performed the local inconsistency test on the closed loops using the node-splitting method; all passed the test, and thus further network meta-analysis was performed. The results of the analysis showed that the lower limit of 95% CI for all closed loops was zero, indicating that there was no significant inconsistency between the closed loops.

The head-to-head comparisons can be seen in Table 2, where different interventions are individually compared with usual care. Overall, resistance exercise (SMD = −1.18, 95% CI [−1.65, −0.71]), aerobic exercise (SMD = −0.72, 95% CI [-0.98, −0.47]), mixed exercise (SMD = −1.06, 95% CI [-1.37, −0.75]), and mind-body exercise (SMD = −0.59, 95% CI [-0.93, −0.26]) were all significantly effective in decreasing depression when compared to the usual care received by the control group. Finally, SUCRA was used to rank the four types of exercise intervention and usual care. As can be seen in Figure 4B, resistance exercise (90.3%) > mixed exercise (81.6%) > aerobic exercise (45.5%) > mind-body exercise (32.6%) > usual care (0%).

**Table 2:** Characteristics of included studies.

**References**	**Country**	**Age**	**Number (T/C)**	**Exercise group**				**Measure**	**Outcome**
				**Type**	**Exercise program duration (week)**	**Frequency (sessions/week)**	**Length (min/sessions)**		
Carter et al. (54)	UK	15.4	36/28	Mixed	6	2	60	CDI-II	①
Hughes (55)	USA	17	14/12	Aerobic	12	1	30–40	CDRS-R	①
Jeong (56)	Korea	16	20/12	Aerobic	12	3	45	SCL-90-R	①③
McCann (57)	USA	NR	15/14	Aerobic	10	2	60	BDI	①
Mohammadi (58)	Iran	NR	80/20	Aerobic	8	3	75	BDI	①
Nabkasorn (59)	Thailand	18.8	21/28	Aerobic	8	5	60	CES-D	①
Roshan (60)	Iran	16.5	12/12	Aerobic	6	3	NR	HAM-D	①
Sadeghi (61)	Iran	20.9	16/14	Aerobic	8	3	45–60	BDI	①
Ma (62)	China	21.4	93/31	Aerobic	12	3	90	SDS	①
Huang (63)	China	18.5	32/32	Mind-body	14	7	25	BDI-II	①
Li (64)	China	NR	18/17	Mixed	8	2	NR	CES-D	①
Jiao (65)	China	21.4	21/23	Aerobic	8	3	NR	SDS	①
Hu (66)	China	18.5	25/25	Aerobic	10	3	NR	SDS/SAS	①
Zhu (67)	China	NR	24/24	Aerobic	8	3	NR	SDS	①
Zheng (68)	China	18.7	40/20	Aerobic	12	2	90	SDS	①
Qiao (69)	China	19.2	62/34	Aerobic	10	1	90	SDS	①
Guan (70)	China	20.5	15/15	Resistance	8	3	NR	CES-D	①
Li (71)	China	20.1	27/26	Aerobic	8	3	NR	SDS	①
Wang (72)	China	NR	16/16	Aerobic	12	NR	90	SCL-90	①
Philippot (73)	Belgium	15.3	26/26	Mixed	6	3–4	60	BDI	①
MacMahon (74)	USA	16.3	32/37	Aerobic	12	3	40	BDI	①
Li (75)	China	21.5	20/20	Mind-body	37	5	60	SDS	①
Li (76)	China	NR	120/120	Mixed	8	5	40	SDS/SAS	①③
Yu (77)	China	18.5	25/19	Mind-body	16	3	60	SDS/SAS	①③
Chen (78)	China	NR	18/18	Mind-body	16	3–4	60	CES-D	①
Li (79)	China	19.9	35/19	Aerobic	12	2–5	NR	SDS	①
Wang (80)	China	22	30/16	Mind-body	12	3–4	40–60	BDI	①
Hu (81)	China	NR	20/20	Mind-body	4	3	NR	SDS	①
Yu (82)	China	18.4	50/19	Mind-body	16	5	60	SDS	①
Li (83)	China	20.5	15/15 15/15	Aerobic Resistance	8	3	30–50	CES-D	①
Zhang (84)	China	18.4	32/32	Mind-body	8	2	90	PHQ-9	①
Yu (85)	China	18.7	43/22	Mind-body	12	3	60	BDI-II	①
Zhao (86)	China	NR	12/12	Mind-body	18	2	90	BDI	①
Noggle (87)	USA	17.2	36/15	Mind-body	10	3	40	POMS	①
Li (88)	China	20.2	15/15	Aerobic	12	5	30–40	CES-D	①
Bonhauser (89)	Chile	15	90/93	Aerobic	40	3	90	HADS	①
Khalsa et al. (90)	USA	16.8	67/34	Mind-body	11	2	30	POMS	①
Velasquez (91)	Colombia	NR	57/57	Mind-body	12	2	120	SDQ	①
Butzer (92))	USA	NR	114/91	Mind-body	16	2	45	RBUMS	①
Costigan (93)	Australia	15.8	19/22 21/22	Aerobic Mixed	8	3	8–10	K-10	①
Goodrich (94)	USA	19.9	15/11	Aerobic	6	3	20–25	RADS	①
Xiaowang (95)	China	NR	7/8 8/8	Aerobic Mind-body	8	2	40	SCL-90	②④
Wenbo Liu (96)	China	20.5	20/20 20/20	Aerobic Mind-body	32	2	90	SCL-90	②④
Wang (97)	China	18.5	22/22 22/22	Aerobic Mixed	9	2	100	SCL-90	②
Yang (98)	China	NR	100/100	Mixed	15	2	90	SDS SAS	②④
Daley (99)	USA	NR	28/30	Aerobic	8	3	30	CDI	②
Hilyer (100)	USA	17	30/30	Mixed	20	3	90	BDI	②
Zhang (101)	China	NR	40/40	Aerobic	NR	NR	NR	SCL-90	②④
Li (102)	China	NR	147/155	Mind-body	12	2	90	SDS	②
Hongfu Liu (103)	China	NR	50/50	Mind-body	12	5	90	POMS	②④
Chen (104)	China	20.8	30/30	Aerobic	8	3	60	POMS	②④
He (105)	China	NR	38/30 22/20	Aerobic Mind-body	12	3	60	POMS	②④
Chen (106)	China	NR	240/240	Aerobic	16	NR	NR	POMS	②④
Gallego (107)	Spain	20.07	42/42	Mixed	8	1	60	DASS-21	②④
Fan (108)	China	19.5	60/60	Resistance	16	3	60	SDS	②
He (109)	China	19.5	60/30 60/30	Aerobic Resistance	16	3	60	SDS	②
Kuttner (110)	USA	NR	14/14	Mind-body	4	NR	60	CDI RCAS	②④
Chu (111)	China	NR	171/57	Mixed	12	3	30–60	SCL-90	②④

Data are those reported by authors and synthesized in this meta-analysis. T experience group, C control group, ① depression of students with depression disorder, ② depression of normal students, ③ anxiety of students with depression disorder, ④ anxiety of normal students, NR, not reported; HAM-D, Hamilton Depression Scale; CDI-II, Children's Depression Inventory 2™; SSRS, the six-item self-report scale; BDI, Beck Depression Inventory; CDRS-R, Children's Depression Rating Scale – Revised; SCL-90-R, Symptom Check List-90-Revision; CES-D, Center for Epidemiological Studies Depression Scale; HADS, Hospital anxiety depression scale; SAS, Self-Rating Anxiety Scale; TAIC, Trait Anxiety Inventory for Children; PHQ-9, Patient Health Questionnaire-9; DIKJ, Depressions Inventar für Kinder und Jugendliche; POMS, profile of mood states; SDQ, the Strengths and Difficulties Questionnaire; RBUMS, The Brunel University Mood Scale; K-10, The Kessler Psychological Distress Scale; RADS, Reynold's adolescent depression scale; RCDS, Reynolds Child Depression Scale; PMSSSF, the Profile of Mood States-Short Form; DASS-21, Depression, Anxiety and Stress Scales; CDI, Children's Depression Inventory; RCAS, Revised Child Manifest Anxiety Scale; BAI, Beck Anxiety Inventory; SCL-90, Symptom Check List-90; SDS, The Zung Self-Rating Depression Scale.

#### 3.5.3. The combined effect of treatment and prevention for depression

The two-dimensional cluster ranking plot in Figure 5 illustrates the overall therapeutic and preventive effects of various interventions on depression in youths. Based on these results, resistance exercise appears to be the most suitable type of exercise for depression in youths, surpassing other exercise types and usual care in terms of both its therapeutic and preventive effects (cluster ranking value: 1914.04).

### 3.6. Moderators of effectiveness: meta-regression and subgroup analysis

Meta-regression (Table 3) showed that there was no statistically significant association between the study characteristics (year, age, country, number of participants, and measurement tools) and intervention effectiveness for depression (*p* > 0.05). Table 4 provides the results of the subgroup analyses. However, the frequency, duration, and length of the interventions were found to be influential features (*p* > 001).

**Table 3:** Random–effects meta regression of SMD according to publication year, age, country, number of participants and measure tool.

**SMD**	**Coefficient**	***p***	**95% CI**	
Year	0.004	0.514	−0.009	0.018
Age	−0.008	0.227	−0.021	0.005
Country	0.049	0.057	−0.001	0.101
Number of participants	0	0.663	−0.001	0.001
Measure tool	0.018	0.299	−0.017	0.054

**Table 4:** Subgroup analysis of SMD according to frequency, session duration and program duration.

**Feature**	**Subgroups**	**Studies**	**SMD**	**95% CI**		**Test for subgroup differences**
Frequency						*p* < 0.001
	≤ 2	21	−0.676	−0.877	−0.475	
	3–4	32	−0.954	−1.105	−0.802	
	≥5	8	−0.887	−1.227	−0.546	
Duration						*p* < 0.001
	≤ 30 min	7	−0.679	−1.006	−0.351	
	30–60 min	14	−0.877	−1.222	−0.532	
	≥60 min	33	−0.836	−0.988	−0.685	
Length						*p* < 0.001
	≤ 6 week	6	−0.512	−0.788	−0.236	
	>6 week	58	−0.869	−0.988	−0.749	

### 3.7. Publication bias

**Figure 6:**
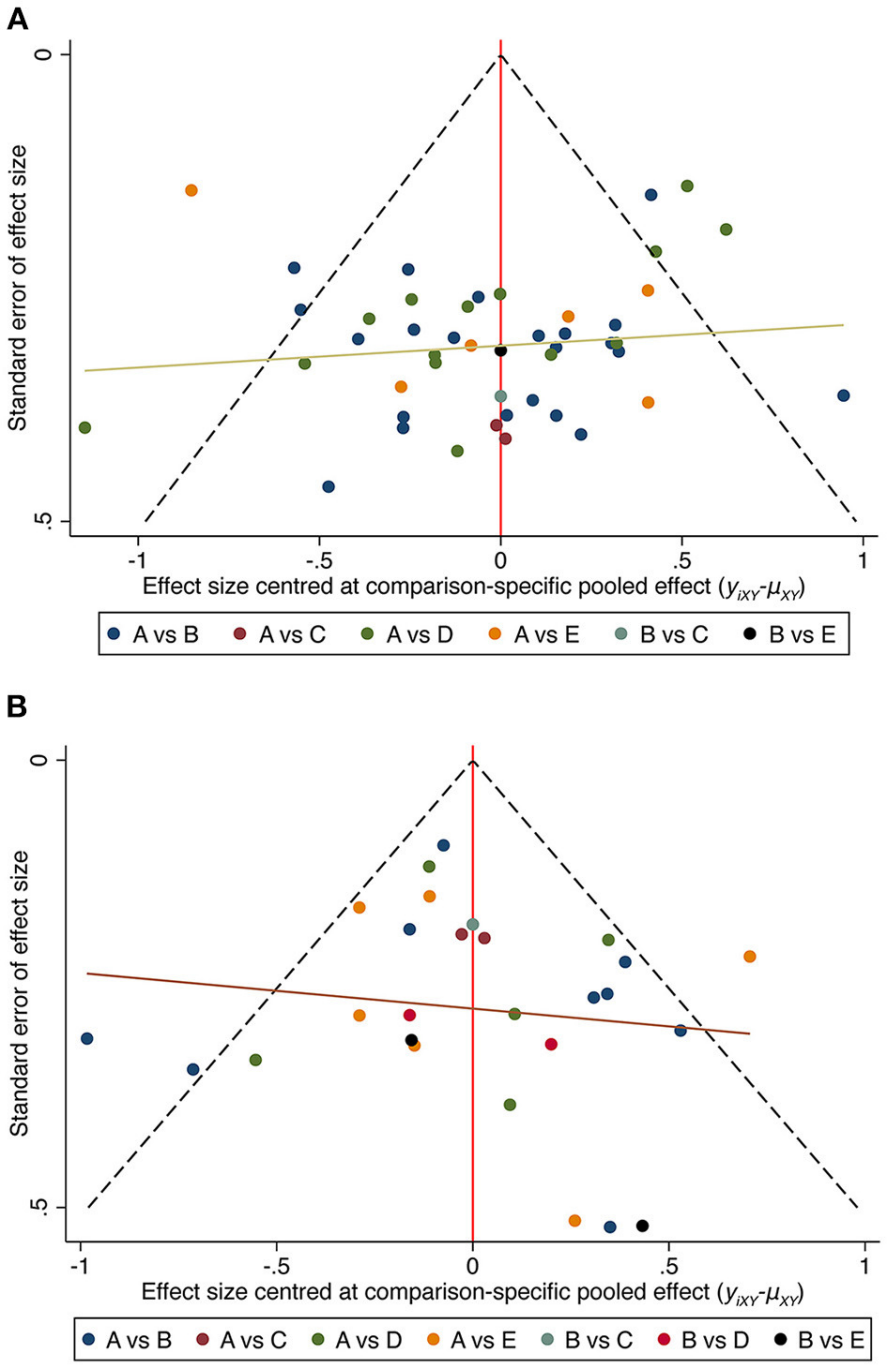
Funnel plots of interventions in depressed youths **(A)** and non-depressed youths **(B)**. A, Usual care; B, Aerobic exercise; C, Resistance exercise; D, Mind-body exercise; E, Mixed exercise.

We conducted publication bias tests for each indicator used to assess the validity of our study's findings. Figure 6 shows that the studies were symmetrically dispersed and evenly distributed on both sides of the midline although several studies fell outside the funnel. The latter fact indicates some impact on the outcomes of the corresponding indicators and suggests the possibility of a publication bias or a small sample effect. Therefore, we conducted a screen-by-exclusion sensitivity analysis and discovered that all the data remained unaltered, suggesting some degree of stability for the study's findings.

## 4. Discussion

The current study employed both meta-analysis and network meta-analysis to investigate the preventive and therapeutic effects of various types of exercise on depression and anxiety in youths. Exercise interventions were found to be significantly superior to usual care in both depressed and non-depressed youths. Further analysis of the combined therapeutic and preventive effects of different types of exercise for depression in youths revealed that resistance exercise is the most advantageous of the various exercise types. This study also found that interventions of 3–4 times per week, with a duration of 30–60 min, and a program length of more than 6 weeks were the most effective for depression interventions in youths.

The first result indicates a significant improvement in anxiety among both non-depressed and depressed youths through exercise intervention, compared to usual care. This is consistent with previous meta-analyses that have evaluated the impact of exercise interventions on anxiety across all age groups, which reported that exercise interventions can be equally as effective as medication interventions (112). Exercise appears to have neurobiological effects on several neural mechanisms that are related to anxiety (113, 114). In particular, mildly psychologically stressful stimuli may result in an attenuated glucocorticoid response, which may manifest as anxious behavior in novel situations. Exercise interventions may mitigate anxiety by influencing the hypothalamic-pituitary-adrenal axis and reducing the glucocorticoid response to stressful stimuli (112, 113). However, it is important to acknowledge that the effects of exercise can differ based on developmental stages related to age. Childhood and adulthood are marked by significant biological, psychological, and psychosocial transformations, as described by Carter et al. (115). Our study addresses the lack of focus on youth populations in prior research and provides evidence supporting the positive impact of exercise on anxiety reduction in youths.

The second finding of our study reveals that a range of exercise types when compared to usual care have significant therapeutic and preventive effects on depression in youths. This suggests that all types of exercise are effective measures for treating and preventing depression, and the findings may be generalizable to both depressed youths and non-depressed youths. Our results are consistent with those of other meta-analyses that investigated the therapeutic effects of exercise on depressed youths (115–117). The positive impact of exercise on depression can be explained through a single theory that appears to be limited in its scope, given the complex interactions involved between psychological and neurophysiological mechanisms (118). Low self-esteem is a predictor of depression in youths (119), and an increase in physical activity is associated with an increase in self-esteem (120, 121). Regular exercise may enhance an individual's self-esteem and alleviate depression by evoking positive feedback from others (20). Furthermore, abnormal oxidative stress is one of the primary causes of depression (122), and exercise can effectively improve oxidative stress (123). Our study not only addresses the lack of research focusing on non-depressed youth populations, but it also employs an innovative use of network meta-analysis to further investigate the effects of different exercise types in treating and preventing depression.

The third result is that resistance exercise is the most effective type of exercise intervention for the comprehensive treatment and prevention of depression in youths. This differs from the results reported by Miller et al. (43), who used the same network meta-analysis method but with older adults as a research object to determine the most effective intervention among different exercise types. The difference in our findings may be due to the physical condition of older adults, who prefer mind-body exercises with low intensity to improve depression (124, 125). The effects of resistance exercise on depression are unlikely to be due to a single, simple process given the complexity of both resistance exercises and mental health (29, 126). A range of biological, psychological, and social factors are plausible reasons for the benefits of exercise in alleviating depression. One possible biological explanation is that resistance exercise, being high-frequency and short-duration, triggers an increase in serum BDNF, promoting central neurotransmitter release and thereby effectively improving depression (127). Furthermore, resistance exercise exerts an anti-inflammatory effect by down-regulating pro-inflammatory markers such as TNF-α and IL-1β mRNA, which have been implicated in depression (128). By contrast, one likely psychological explanation is self-efficacy, a central concept in Bandura et al. (129) social cognitive theory, as successful experiences can increase an individual's self-efficacy and thereby decrease the likelihood of depression and anxiety (119). Specifically, strength is a very significant indicator because increasing strength resulting from resistance exercise is clearly perceptible, whereas improvements in speed and endurance may be less noticeable (130). Consistent with this, prior studies have found that increasing strength is positively related to self-efficacy (131) and inversely related to depression (132). Therefore, resistance exercise can improve inhibition deficits and insufficient pleasure by producing more psychologically positive feedback and counteracting negative emotions (133–135). This study bridges a research gap involving youth populations and includes depressed and non-depressed youths to consider the therapeutic and preventive effects of different types of exercise, supporting the use of resistance exercise as the optimal type of exercise intervention in both depressed and non-depressed youths.

The fourth result shows that the frequency, duration, and length of the interventions are the moderators of their effectiveness. First, we found that interventions carried out 3–4 times per week were more effective in improving depression in youths when compared to interventions conducted <2 times per week or more than 5 times per week. This finding agrees with those of Giles et al. (136), who used the exercise intervention to investigate the improvement of major depressive disorder in youths. Regular exercise can provide a sense of routine and structure, and these may be particularly beneficial for individuals with depression, who may struggle with feelings of disorganization or lack of purpose (137). However, too much exercise may lead to physical and mental health problems such as overuse injuries, burnout, and increased stress (138). Second, exercise interventions of 30–60 min duration were associated with greater improvements in depression than shorter sessions. This finding is consistent with previous meta-analyses that have evaluated the impact of exercise interventions in people without clinical depression across all age groups (139). One possible reason for this is that longer exercise sessions may lead to a greater increase in body temperature, which can stimulate the production of hormones and neurotransmitters involved in mood regulation (140). However, research also suggests that the number of endorphins released during exercise is proportional to the duration of the exercise (141). Thus, shorter exercise duration in a session, such as <30 min, may not provide enough stimulus to trigger a significant release of endorphins, and this could limit the mood-enhancing effects of exercise (142). Meanwhile, longer exercise durations, such as more than 1 h, may be more difficult to maintain over time due to scheduling conflicts, time constraints, or other factors, and this can lead to a decrease in compliance (143). Third, interventions that were longer in terms of the overall length of the exercise program were found to be more effective in decreasing depression than those that were shorter in length. Studies have shown that regular exercise can lead to physiological adaptations that improve cardiovascular health, increase the release of endorphins, and reduce stress levels (144). However, these adaptations may take some time to occur. Meanwhile, exercise programs that involve group or supervised sessions may provide opportunities for social support, which has been shown to be a protective factor for mental health (145). Longer exercise programs may provide more opportunities for individuals to develop supportive relationships with other participants or with exercise professionals (146), and this could, in turn, contribute to greater improvements in depression. This study bridges a gap in the lack of research focusing on youth populations and depression to consider the specific exercise setting during the intervention.

Overall, resistance exercise interventions with a frequency of 3–4 times per week, session duration of 30–60 min, and more than 6 weeks in length were associated with the greatest improvements in depression. This suggests that exercise interventions with these specific characteristics may be more effective in reducing depression than interventions with different exercise types, frequencies, session durations, or program lengths. This highlights the importance of carefully considering the design and implementation of exercise interventions for individuals with depression and suggests that interventions with these specific characteristics may be a promising avenue for future research and clinical practice.

### 4.1. Practical implications

This study has significant practical implications for schools regarding the development of physical education curricula for youths that would promote the prevention and treatment of depression. It is well-established that patients with mild to moderate depression often refuse treatment due to the associated stigma and high costs and side effects of medication, which may exacerbate their depression and increase the likelihood of recurrence and suicidal behavior (147). The results of this study rank different types of exercise in terms of their efficacy for the treatment and prevention of depression, offering valuable guidance for school psychological counselors and administrators. As youths spend a significant portion of their social lives in school, the school environment presents a unique opportunity for effective intervention in stress and anger management. Moreover, schools possess the necessary infrastructure for implementing both prevention and treatment programs. Therefore, the inclusion of resistance exercises in daily physical education curricula may not only prevent depression in youths but also circumvent the associated stigma of treatment for depressed youths. Such an approach promises greater compliance and improved treatment outcomes.

### 4.2. Limitations and future directions

We recognize that this study has several limitations. (1) We only combined all relevant studies and explored the differences of effects in the treatment or prevention of depression between different exercise types by a network meta-analysis. Future studies should further explore the mechanisms of their effects. (2) There was a lack of investigation of youths' adherence to different types of exercise interventions. Future research should assess adherence to different exercise interventions in order to determine the optimal exercise type under the consideration of a combined effect. (3) The majority of studies included in this analysis were of low quality, relying on self-reported outcomes measured through scales, and only a few provided clear details on blinded outcome assessment and allocation concealment. This is in line with previous meta-analyses conducted by Wang et al. (124), McDermott et al. (148), and Larun et al. (149). Despite the efforts made in this study to enhance the robustness of the results by including more eligible studies, there is still a need for more studies in the field that adhere to the highest methodological standards and use clinical diagnosis. (4) The clinical presentation of somatic disturbances, characterized by decreased cognitive function, diminished interest, and impaired motor activity, is commonly observed in patients with moderate to severe depression (150). Consequently, individuals with depression have significant differences in their physical motor capabilities compared to those without (151). Specifically, depressed youths exhibit a heightened susceptibility to fatigue during physical activity (152), and a negative correlation exists between youths' motor proficiency and physical fitness and their depression severity (153). Therefore, there is an urgent need for comprehensive investigations and personalized interventions to facilitate exercise participation among depressed youths. (5) Some of the studies included in our analysis did not report key data or used different outcome measures, limiting our ability to perform a more comprehensive meta-analysis. Therefore, we must emphasize the importance of standardized reporting in future studies to facilitate more comprehensive meta-analyses. Standardization of outcome measures and data reporting will increase the quality and comparability of research findings, ultimately leading to more robust and generalizable conclusions. (6) While this study focused on the immediate effects of exercise interventions, it is possible that the benefits may diminish over time without continued support or reinforcement. Future studies could examine the impact of follow-up interventions or ongoing support on long-term effectiveness. Additionally, exploring any factors that may influence adherence to follow-up interventions could also be valuable for optimizing sustainability. Despite these limitations, this study is the first to explore the treatment and prevention effects of different types of exercise for depression in youths.

## Data availability statement

The original contributions presented in the study are included in the article/Supplementary material, further inquiries can be directed to the corresponding author.

## Author contributions

ZS and YiZ conceived the study design, collected, analyzed the data, and drafted the manuscript. GL and CL participated in the study design, collected, and analyzed data. YuZ and JG assisted in revising the manuscript, reviewed the first, and final versions of the manuscript. All authors contributed to this article and agreed to the submitted version of the manuscript.
