# Corrigendum: Comparing the efficacy of different types of exercise for the treatment and prevention of depression in youths: a systematic review and network meta-analysis

**DOI:** 10.3389/fpsyt.2023.1304302

**Published:** 2023-10-10

**Authors:** Yihan Zhang, Geng Li, Chengzhen Liu, Jinliang Guan, Yuantong Zhang, Zifu Shi

**Affiliations:** ^1^School of Educational Science, Hunan Normal University, Changsha, China; ^2^College of Physical Education, Hunan Normal University, Changsha, China

**Keywords:** depression, anxiety, exercise, youth, treatment, prevention

In the published article, an author name was incorrectly written as Zhifu Shi. The correct spelling is Zifu Shi.

The authors apologize for this error and state that this does not change the scientific conclusions of the article in any way. The original article has been updated.

