# Supplementary material for: Comparing the efficacy of different types of exercise for the treatment and prevention of depression in youths: a systematic review and network meta-analysis
Source: Front Psychiatry. 2023 Jun 2;14:1199510. doi: 10.3389/fpsyt.2023.1199510 (PMC10272399; doi:10.3389/fpsyt.2023.1199510)
Supplement: Supplementary file 1 [file Data_Sheet_1.docx]

Supplementary Material

Article Title

First Author*, Co-Author, Co-Author

*** Correspondence:** Corresponding Author: email@uni.edu

# Supplementary Figures and Tables

## Supplementary Figures

**

**

**Supplementary Figure 1.** Risk of bias summary.


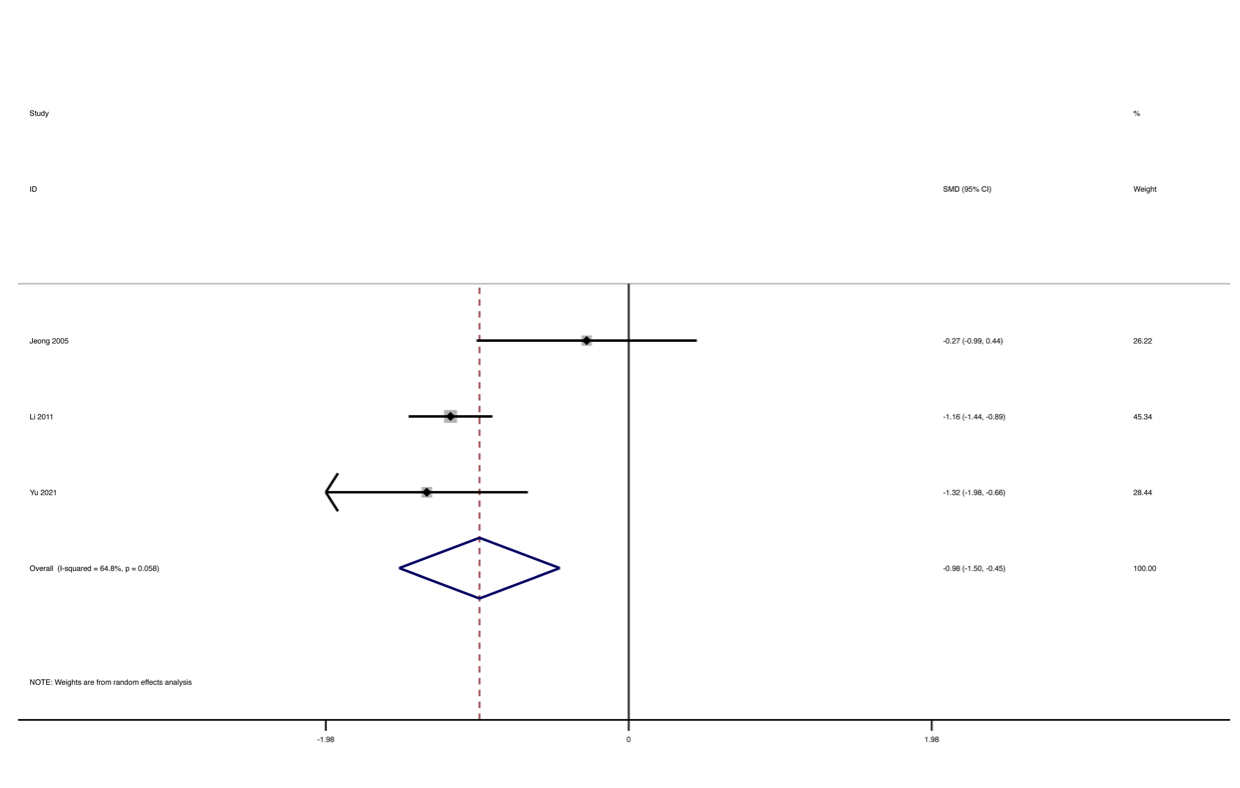


**Supplementary Figure 2.** Forest plot of anxiety for depressed youths.


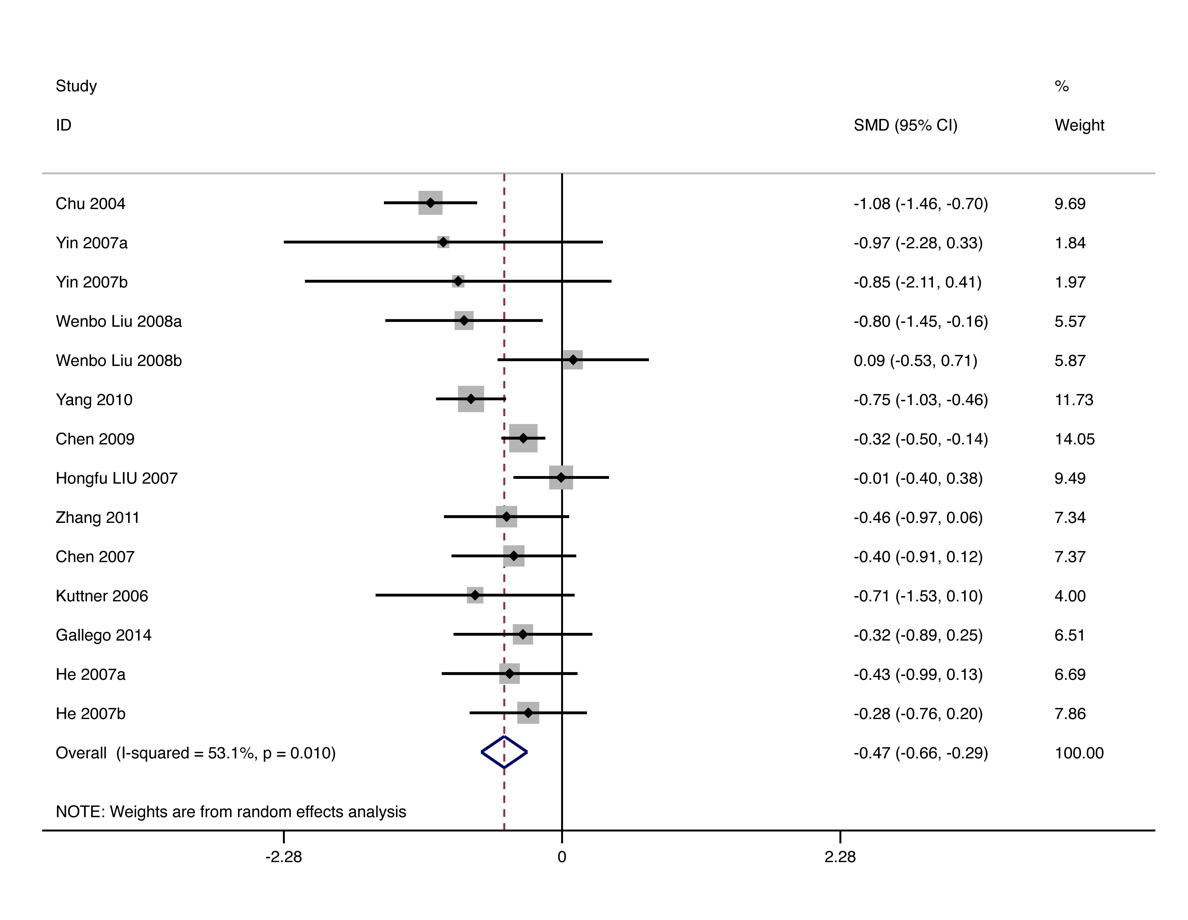


**Supplementary Figure 3.** Forest plot of anxiety for non-depressed youths.





**Supplementary Figure 4.** Sensitivity analysis of depression for depressed youths.





**Supplementary Figure 5.** Sensitivity analysis of depression for non-depressed youths.

## Supplementary Tables

**Supplementary Table 1.** Search strategy.

(Depress∗ [Title/Abstract] OR “affective symptom∗” [Title/Abstract] OR “affective disorder∗ ” [Title/Abstract] OR “mood disorder∗ ” [Title/Abstract]) AND (youth∗ [Title/Abstract] OR adolesc∗ [Title/Abstract] OR pubert∗ [Title/Abstract] OR girl* [Title/Abstract] OR boy* [Title/Abstract] OR school* [Title/Abstract] OR teen*[Title/Abstract] OR underag* [Title/Abstract] OR under-ag* [Title/Abstract]) AND (exercis∗ [Title/Abstract] OR sport∗ [Title/Abstract] OR “physical activity” [Title/Abstract] OR “physical exertion” [Title/Abstract] OR “physical training” [Title/Abstract] OR “physical education” [Title/Abstract] OR running [Title/Abstract] OR jogging [Title/Abstract] OR walking [Title/Abstract] OR bicycling [Title/Abstract] OR swimming [Title/Abstract] OR “strength training” [Title/Abstract] OR “resistance” [Title/Abstract]) OR “mind-body” [Title/Abstract]) OR “aerobic” [Title/Abstract]) weightlift* [Title/Abstract] OR powerlift* [Title/Abstract] OR "musc* [Title/Abstract] OR strength* [Title/Abstract] OR football* [Title/Abstract] OR soccer* [Title/Abstract] OR volleyball*[Title/Abstract] OR basketball* [Title/Abstract] OR baseball* [Title/Abstract] OR handball* [Title/Abstract] OR cricket* [Title/Abstract] OR rugby* [Title/Abstract] OR tennis* [Title/Abstract] OR badminton* [Title/Abstract] OR stretching* [Title/Abstract] OR gymnastic [Title/Abstract])

**Supplementary Table 2.** Baseline Depression Severity Categories.

| **Scale** | **Depression Severity Category** | | | |
| --- | --- | --- | --- | --- |
|  | **Sub-threshold** | **Threshold** | | |
|  |  | **Mild** | **Moderate** | **Severe** |
| BDI  (BDI & BDI-II)  (Beck *et al.* 1996; Beck *et al.* 1988) | 0 – 12 | 13 – 19 | 20 – 29 | ≥ 30 |
| CDI  (Friedberg, 2011) | 0 – 12 | ≥ 13 | Not specified | Not specified |
| HAMD  (Hamilton, 1960) | 0 – 7 | 8 – 13 | 14 – 18 | ≥ 19 |
| CES-D  (Radloff, 1977) | 0 – 15 | 16 – 26 | Not specified | Not specified |
| SDS  (Zung *et al.* 1965) | 0 – 53 | 53 –62 | 63 –72 | ≥ 73 |

**References**

Beck, A. T., Steer, R. A., & Brown, G. (1996). Beck depression inventory–II. *Psychological assessment*.

Beck, A. T., Steer, R. A., Ball, R., & Ranieri, W. F. (1996). Comparison of Beck Depression Inventories-IA and-II in psychiatric outpatients. *Journal of personality assessment*, 67(3), 588-597.

Friedberg, R. D., & Sinderman, S. A. (2011). CDI score in pediatric psychiatric inpatients: A brief retrospective static group comparison. *Depression Research and Treatment*, 2011.

Hamilton, M. (1960). The Hamilton Depression Scale—accelerator or break on antidepressant drug discovery. *Psychiatry*, 23(1), 56-62.

Radloff, L. S. (1977). The CES-D Scale: A self-report depression scale for research in the general population. *Applied Psychological Measurement*, 1(3), 385–401. <https://doi.org/10.1177/014662167700100306>

Zung, W. W., Richards, C. B., & Short, M. J. (1965). Self-rating depression scale in an outpatient clinic: further validation of the SDS. *Archives of general psychiatry*, 13(6), 508-515.

**Supplementary Table 3.** Definition for variables.

1. Exercise: refers to planned, structured, repetitive, and purposeful physical activities aimed at improving or maintaining one or more physical fitness components (Caspersen et al., 1985).
2. Depression: refers to a mental health disorder characterized by feelings of sadness, hopelessness, and loss of interest in activities (APA, 2013).
3. Anxiety: refers to a mental health disorder characterized by feelings of worry, nervousness, or unease (APA, 2013).
4. aerobic exercise: refers to a physical exercise performed under conditions of adequate oxygen supply (Mersy, 1991).
5. mind-body exercise: refers to the interaction between the brain, mind, and body behavior (La forge, 1997).
6. mixed exercise: refers to sports that combine more than two types of exercise.
7. resistance exercise: refers to training to increase muscular strength through active, assisted active and resistance exercises (Howley, 2001).

Reference

Caspersen, C. J., Powell, K. E., & Christenson, G. M. (1985). Physical activity, exercise, and physical fitness: definitions and distinctions for health-related research. *Public health reports* (Washington, D.C. : 1974), 100(2), 126–131.

American Psychiatric Association, DSM-5 Task Force. (2013). Diagnostic and statistical manual of mental disorders: DSM-5™ (5th ed.). *American Psychiatric Publishing*, Inc.. <https://doi.org/10.1176/appi.books.9780890425596>

Mersy, D. J. (1991). Health benefits of aerobic exercise. *Postgraduate medicine*, 90(1), 103-112.

La Forge R. (1997). Mind-body fitness: encouraging prospects for primary and secondary prevention. *The Journal of cardiovascular nursing*, 11(3), 53–65. https://doi.org/10.1097/00005082-199704000-00006

Howley, E. T. (2001). Type of activity: resistance, aerobic and leisure versus occupational physical activity. *Medicine and science in sports and exercise*, 33(6 Suppl), S364-9.

**Supplementary Table 4.** Assumption in this study.

There are no specific assumptions mentioned in this study. However, the study assumes that different types of exercise can have different therapeutic and preventive effects on depression and anxiety in youths. It also assumes that exercise can have a positive impact on anxiety levels in both depressed and non-depressed youths. Additionally, the study assumes that combining certain types of exercise can maximize their therapeutic and preventive effects in youths. These assumptions form the basis of the research questions and the study's objective to develop more effective exercise interventions for depression and anxiety in youths.

**Supplementary Table 5.** Simplifications in this study.

(1) SMD: standardized mean difference

(2) CI: confidence interval

(3) SUCRA: the surface under the cumulative ranking score

(4) PRISMA: Preferred Reporting Items for Systematic Reviews and Meta-Analyses

(5) PICOS: Population, Intervention, Comparison, Outcome, and Study design

(6) RCTs: randomized controlled trials

(7) IF: inconsistency factor

(8) NMA: network meta-analysis

(9) APA: American Psychiatric Association
